# Supplementary material for: Association Between Dietary Tomato Intake and Blood Eosinophil Count in Middle-Aged and Older Japanese Individuals: A Population-Based Cross-Sectional Study
Source: Nutrients. 2025 Nov 3;17(21):3467. doi: 10.3390/nu17213467 (PMC12609217; doi:10.3390/nu17213467)
Supplement: Supplementary file 1 [file nutrients-17-03467-s001.zip › 251005TableS1S2.pdf]

Table S1. A multivariable logistic regression analysis results for elevated blood eosinophil counts (Model 3).

	OR	95% CI		p-value
Men, vs. women	1.689	0.999	2.855	0.050
Age, per +1 year	0.992	0.973	1.011	0.419
BMI, per +1 kg/m^2	1.051	0.984	1.122	0.137
Energy intake, per +1 kcal	1.000	1.000	1.000	0.807
Tomato intake, per +10 g/day	0.877	0.792	0.980	**0.020**
Drinking	1.413	0.879	2.271	0.153
Smoking	1.998	1.214	3.291	**0.007**
Asthma	2.852	1.058	7.690	**0.038**
PRS for asthma	12.896	1.241	133.974	**0.032**

A forced entry binary multivariable logistic regression model was used (n = 535). OR, odds ratio; CI, confidence interval; BMI, body mass index; PRS, polygenic risk score. The p-values that are <0.05 are highlighted in bold.

Table S2. Association between vitamin consumptions and elevated blood eosinophil count.

	OR	95% CI		p-value
Retinol equivalent, +1μg/day	1.000	0.999	1.000	0.394
Carotene equivalent., +1μg/day	1.000	1.000	1.000	0.364
Folate intake, +1μg/day	1.000	0.998	1.001	0.565
Vitamin C intake, +1mg/day	0.998	0.995	1.002	0.317

Covariates：age, sex, daily energy intake, body mass index, drinking habits, current smoking status and asthma

OR, odds ratio; CI, confidence interval.
