# Supplementary figures and images for: Association Between Dietary Tomato Intake and Blood Eosinophil Count in Middle-Aged and Older Japanese Individuals: A Population-Based Cross-Sectional Study
Source: Nutrients. 2025 Nov 3;17(21):3467. doi: 10.3390/nu17213467 (PMC12609217; doi:10.3390/nu17213467)

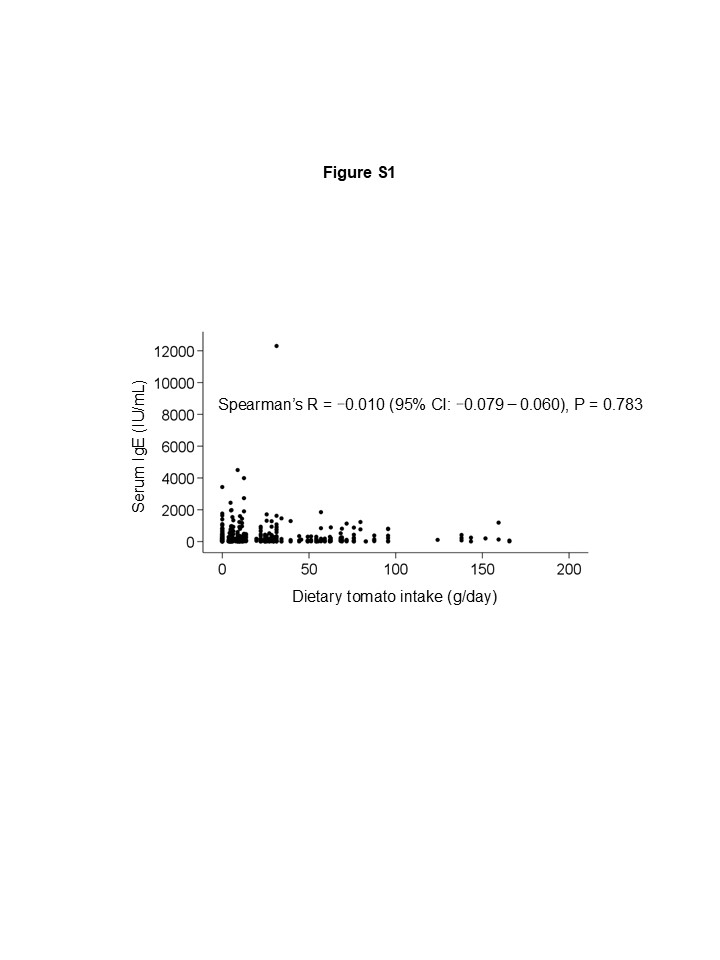

Supplement: Supplementary file 1 [file nutrients-17-03467-s001.zip › Figure S1_nutrients.jpg]
